# Reduction of peak plantar pressures in patients with peripheral neuropathy: an evaluation of the DH Pressure- Relief™ Shoe

**DOI:** 10.1186/1757-1146-4-S1-O38

**Published:** 2011-05-20

**Authors:** Anita Raspovic, Karl Landorf, Jana Gazarek, Megan Stark

**Affiliations:** 1 Department of Podiatry and Musculoskeletal Research Centre, La Trobe University, Melbourne, Victoria, 3086, Australia; 2 Ms Jana Gazarek, Northern Health Podiatry Department, Bundoora Extended Care Centre, Melbourne, Victoria, 3083, Australia; 3 Private podiatrist, Melbourne, Australia

## Background

Off-loading of plantar pressure is a key intervention strategy to prevent the formation of or to heal existing neuropathic plantar ulcers in diabetes. Walking casts (e.g. total contact cast) that reduce plantar pressures are currently considered the gold-standard for treating such neuropathic ulcers. However, alternative methods for off-loading that are as effective, but cheaper and easier to use are continually being sought. The aim of this study was to evaluate the effectiveness of the DH Pressure- Relief™ Shoe in off-loading high pressure areas in the neuropathic foot in diabetes.

## Methods

A repeated measures design was used. Sixteen participants with diabetic peripheral neuropathy were recruited and three footwear conditions were evaluated in a randomised order: a canvas shoe (the control), the participants’ own shoe, and the DH shoe. The primary outcome was peak plantar pressure measured using the PedarX^®^ mobile in-shoe system between the 3 footwear conditions.

## Results

Data analysis was carried out on 14 out of the 16 participants because two participants could not complete data collection as planned. The mean peak pressure values in KPa (±SD) for each condition were: control shoe 315.9 (±140.7), standard shoe 273.0 (±127.1) and DH shoe 155.4 (±89.9). There was a statistically significant difference (*p*=0.001) in peak plantar pressure between the DH shoe compared to both the control shoe and participants standard shoe. The DH shoe decreased plantar pressures by 51% compared to the control shoe and by 43% compared to standard shoe.

## Conclusions

The DH Pressure-Relief Shoe™ reduced plantar pressures significantly more that the other two shoe conditions. The DH Pressure-Relief Shoe™ may be a useful alternative to current off-loading modalities used in the clinical management of diabetic foot ulceration. However, clinical trials are needed to ensure they are useable and safe for patients in everyday activities.

